#  Epigenetics impacts upon prognosis and clinical management of choroid plexus tumors

**DOI:** 10.1007/s11060-020-03509-5

**Published:** 2020-04-28

**Authors:** Christian Thomas, Katie Metrock, Uwe Kordes, Martin Hasselblatt, Girish Dhall

**Affiliations:** 1 Institute of Neuropathology, University Hospital Münster, Münster, Germany; 2 Division of Pediatric Hematology, Oncology, and Blood & Marrow Transplantation, University of Alabama at Birmingham, Birmingham, USA; 3 Department of Pediatric Hematology and Oncology, University Medical Center Hamburg- Eppendorf, Hamburg, Germany

**Keywords:** Choroid plexus tumor, TP53, DNA methylation profiling, 850 k, Copy number alterations, Prognosis, Treatment

## Abstract

**Purpose:**

Choroid plexus tumors comprise of choroid plexus papilloma (CPP, WHO grade I), atypical choroid plexus papilloma (aCPP, WHO grade II) and choroid plexus carcinoma (CPC, WHO grade III). Molecular events driving the majority of choroid plexus tumors remain poorly understood. Recently, DNA methylation profiling has revealed different epigenetic subgroups.

**Methods:**

Comprehensive review of epigenetic profiles of choroid plexus tumors in the context of histopathological, genetic, and clinical features.

**Summary:**

DNA methylation profiling segregates choroid plexus tumors into three distinct epigenetic subgroups: supratentorial pediatric low-risk choroid plexus tumors (CPP and aCPP), infratentorial adult low-risk choroid plexus tumors (CPP and aCPP), and supratentorial pediatric high-risk choroid plexus tumors (CPP and aCPP and CPC). Epigenetic subgrouping provides additional prognostic information in comparison to histopathological grading.

**Conclusions:**

Epigenetic profiling of choroid plexus tumors can be used for the identification of patients at risk of recurrence and is expected to play a role for treatment stratification and patient management in the context of future clinical trials.

## Introduction

Choroid plexus tumors are rare neoplasms derived from the choroid plexus epithelium and represent 0.2% of all central nervous system (CNS) neoplasms, but up to 20% of brain tumors arising throughout the first year of life [1]. According to the World Health Organization (WHO) classification, choroid plexus tumors can be divided into choroid plexus papilloma (CPP, WHO grade I), atypical choroid plexus papilloma (aCPP, WHO grade II) and choroid plexus carcinoma (CPC, WHO grade III) [1].

CPP is a benign papillary neoplasm closely resembling non-neoplastic choroid plexus tissue with a single layer of monomorphic epithelial cells covering a highly vascularized stroma. Mitotic activity is absent or very low (< 2 mitoses per 10 high-power fields). CPP has an average annual incidence rate of 0.3 per 1,000,000 and outnumbers CPC by a factor of 5:1 [2, 3]. The median age at diagnosis is four years for CPP and one year for both aCPP and CPC with a majority of patients diagnosed before five years of age [4]. CPPs predominantly arise in the atrium of the lateral ventricle in children [5] and the posterior fossa in adults [6]. Less frequently, CPPs occur in the third ventricle, whereas extraventricular locations (such as the sellar region [7] or brain parenchyma [8]) are exceptional. The majority of patients with CPP experience excellent outcomes following a gross total resection with a 5-year overall survival (OS) rate of 90–100% [9, 10], although distant metastases, either at the time of diagnosis or up to several years after initial diagnosis, have been described [11]. In a retrospective analysis, recurrence rate of 6% among 103 patients with CPP and malignant transformation to CPC in only 1 patient was described [12].

In contrast, CPC is a highly malignant neoplasm most commonly occurring in the lateral ventricles. At least four of the following five histological features are required for a diagnosis of CPC: brisk mitotic activity (> 5 mitoses per 10 high-power fields), nuclear pleomorphism, high cellularity, blurring of the papillary growth pattern, and areas of necrosis. Despite aggressive treatment protocols, including surgical resection and combination of chemotherapy and radiation therapy, the clinical behavior of CPC is variable with 5-year OS rates between 56% and 64% [9, 10] with many long-term survivors suffering from neurocognitive deficits [13]. While metastatic disease is rare in CPP, 21% of patients with CPC present with metastatic disease at diagnosis [14].

For decades, choroid plexus tumors have been classified as either CPP or CPC, but such a binary distinction was difficult, as some CPP display one or even several atypical histological features [2]. On multivariate analysis of a large cohort comprising 124 CPPs affecting children and adult patients, increased mitotic activity, defined as ≥ 2mitoses/10 high-power fields (area of view 0.23 mm^2^), was the only clinicopathological feature associated with a higher probability of recurrence [15]. This observation was then introduced in the 2007 WHO definition of aCPP, i.e., CPP with increased mitotic activity as an intermediate risk group. However, in a subsequent multi-institutional study conducted by SIOP, aCPP did not have significantly worse PFS when compared with CPP [14], raising the possibility that increased mitotic activity might not have an adverse prognostic effect in younger children. Subsequently, another study comprising 149 pediatric patients with CPP and aCPP demonstrated that the prognostic value of increased mitotic activity was restricted to children > 3 years old [16]. Underlying biological factors explaining this effect remain elusive but could be related to a milieu favoring proliferative activity in the choroid plexus throughout the first years of life.

## Biology of choroid plexus tumors

Although the majority of choroid plexus tumors occur sporadically, CPCs are strongly associated with Li-Fraumeni syndrome (LFS), a classic cancer predisposition disorder caused by germline mutations of the *TP53* tumor suppressor gene [17]. The frequency of *TP53* germline mutations in patients with CPC ranges from 26% [18] to 44% [19]. Cases of constitutional mosaicism are also on record [20]. Due to a founder effect, *TP53* germ-line mutations (p.R337H) are highly prevalent in southern Brazil (up to 63% of the patients with CPC) [21]. Sanger sequencing of a cohort of 64 choroid plexus tumors revealed somatic *TP53* mutations in 50% of CPCs but only 5% of CPPs [19], while other studies reported somewhat lower (36%) [22] or higher (60%) [18] rates of somatic *TP53* mutations in CPC. Somatic *TP53* mutations or p53 protein accumulation (as a surrogate marker) have been linked to reduced overall and event-free survival in patients with CPC [19]. Also, combining *TP53* sequencing results with allele-specific copy-number status of chromosome 17, a higher number of mutated *TP53* copies has been associated with a worse outcome [18]. Thus, in a clinical setting, *TP53* mutation status may be helpful in risk stratification of CPCs and a thorough family history to rule out Li-Fraumeni syndrome should be obtained. Other rare genetic diseases associated with CPTs include Aicardi syndrome; X-linked genetic disorder characterized by agenesis of the corpus callosum, chorioretinal lacunae, infantile spasms and increased risk of developing (sometimes multifocal) CPPs [23]. Cases of CPP have also been reported in the context of Hyomelanosis of Ito with translocation (X;17) [24] and CPC in a family with Lynch syndrome (hereditary nonpolyposis colorectal cancer) with *MSH6* mutation [25]. Despite the known association with genetic syndromes, large scale sequencing studies of CPPs and aCPPs are lacking, and whole genome sequencing (WGS) performed in three *TP53*-wildtype and one *TP53*-mutated CPC did not reveal any other recurrent mutation [26].

On a structural level, choroid plexus tumors are characterized by a high degree of chromosomal imbalances. Early cytogenetic analyses have revealed hyperdiploid chromosome sets in CPPs [27, 28] and mainly hypodiploid genomes in CPCs [29, 30]. Using array comparative genomic hybridization (CGH), Rickert et al.. have correlated chromosomal imbalances of 34 CPPs and 15 CPCs with clinical follow-up [31]. In that study, gains of chromosome 9p and losses of chromosome 10q were associated with longer survival in CPC. In a more recent study comprising 26 CPCs, loss of chromosome 12q was significantly associated with shorter survival on multivariate analysis, taking into account extent of resection and administration of radiotherapy [32]. Other studies involving larger cohorts confirmed that CPPs and aCPPs are mainly characterized by whole chromosomal gains, whereas CPCs mainly display chromosomal losses [22, 26, 33]. Interestingly, Merino et al.. were able to separate CPCs into two distinct groups of hypo- and hyperdiploid CPCs and allele-specific analyses pinpoint that most hyperdiploid CPCs are characterized by acquired uniparental disomy (aUPD) of several chromosomes with chromosome 17 (containing *TP53* on 17p13.1) being most affected by this alteration [18]. In summary, chromosomal imbalances are very frequent in choroid plexus tumors, but it remains to be investigated if these alterations are tumor driving events or rather represent a passenger effect during tumor growth and progression.

On gene expression level, unsupervised clustering clearly segregates CPC from CPP and aCPP [18, 22]. With the exception of cell cycle related genes that appear to be overrepresented in aCPP compared to CPP, CPP and aCPP in very young children share similar gene expression signatures [18, 22]. Given that aCPP is associated with recurrence only in older children (≥ 3y) and adults [16], underlying biological factors explaining these age-dependent prognostic differences in aCPP remain to be determined.

In summary, molecular genetic events driving the majority of choroid plexus tumors remain poorly understood. Recently, however, DNA methylation profiling revealed three epigenetically distinct and clinically relevant subgroups of choroid plexus tumors [18, 33].

## Epigenetic subgroups of choroid plexus tumors

Epigenetic dysregulation including alterations of DNA methylation has long been recognized as an important factor contributing to tumorigenesis and tumor maintenance. In particular, specific changes of DNA methylation in small genomic regions were shown to serve as promising targets for the development of powerful prognostic and predictive biomarkers such as MGMT methylation in malignant glioma [34]. On the other hand, global DNA methylation alterations occur due to large somatically acquired changes [35], while retaining characteristics that reflect the cell of origin [36]. Taking advantage of such patterns, methylation-based tumor classification has emerged as a highly robust and reproducible tool even in small biopsies and poor-quality samples to improve diagnostic accuracy and identification of clinically relevant subgroups throughout the last years, including choroid plexus tumors [33, 37, 38]. Merino et al. have shown that CPPs and aCPPs of pediatric patients share epigenetic signatures and segregate from most CPCs on DNA methylation profiling [18]. In another series of 92 choroid plexus comprising 29 CPPs, 32 aCPPs and 31 CPCs of pediatric and adult patients, DNA methylation profiling revealed three epigenetically distinct and clinically relevant subgroups [33]. As summarized in Table 1, tumors of methylation cluster 1 comprised CPPs and aCPPs characterized by young age and mainly supratentorial location, while methylation cluster 2 comprised mainly infratentorial CPP and aCPPs of adult patients. In contrast, all 31 CPCs clustered with methylation cluster 3 and were characterized by young age and mainly supratentorial location. Importantly, 14 of 32 aCPPs (44%) and 5 of 29 CPPs (17%) shared epigenetic similarities with CPCs and also clustered with methylation cluster 3. The majority of CPPs and aCPPs were encountered within methylation cluster 1 and methylation cluster 2. Both of these clusters differed significantly by age and tumor location. Both methylation clusters also differed on the genetic level, with gains of chromosome 5 and losses of chromosome 21q being more frequently encountered in methylation cluster 2.

**Table 1:** Nomenclature, histopathology and clinical features of epigenetic choroid plexus tumor subgroups

Designation CNS tumor classifier [37]	Plexus tumor, subclass pediatric A	Plexus tumor, subclass adult	Plexus tumor, subclass pediatric B
Designation in [33]	Cluster 1	Cluster 2	Cluster 3
Histopathology	CPP and aCPP	CPP and aCPP	CPP, aCPP and CPC
Age group	Pediatric	Adult	Pediatric
Tumor location	Supratentorial	Infratentorial	Supratentorial
Clinical outcome	Low risk	Low risk	High risk

While outcome in methylation cluster 1 and methylation cluster 2 was favorable, prognosis of patients in methylation cluster 3 was worse. In this cluster, which contained all CPCs and all p53-positive tumors examined, a total of eight tumor-related deaths occurred, whereas none of the patients in methylation cluster 1 and methylation cluster 2 succumbed to their disease. On multivariate analysis taking into account WHO grade, age, location, copy-number alterations, and p53 status as co-factors, WHO grade (but not methylation clustering) was the only independent predictor of OS whereas the WHO grade and p53 status remained the only predictors of PFS. Analyzing the prognostic role of methylation clustering according to the WHO grade revealed that all recurrences observed in aCPPs occurred in methylation cluster 3, whereas none of the aCPPs of the low-risk clusters (cluster 1 and cluster 2) recurred. Taken together, these data suggest that DNA methylation profiling might be especially useful (and also superior to histopathology) for identifying aCPPs at increased risk of recurrence.

**Fig. 1:**
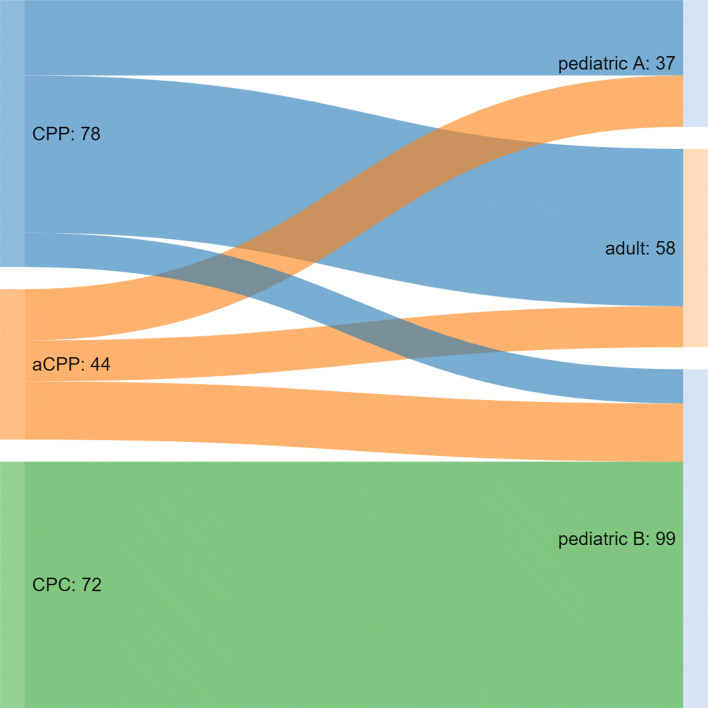
Histopathology and epigenetic subgroups in choroid plexus tumors. Riverplot showing DNA methylation-based subgroup assignment for each histological subtype. Epigenetic classification of 194 choroid plexus tumors (78 CPP, 44 aCPP and 72 CPC) showing that CPP and aCPP branch into all three epigenetic subgroups, while CPC cases are uniformly associated with methylation class pediatric B. Numbers represents number of assigned samples (N)

DNA methylation profiling increasingly becomes part of routine neuropathology diagnostics [39] and is available as a web-based platform (www.molecularneuropathology.org). As summarized in Table 1, the CNS Tumor Classifier methylation classes “plexus tumor, subclass pediatric A” and “plexus tumor, subclass pediatric B” correspond to methylation cluster 1 and 3, respectively, whereas “plexus tumor, subclass adult” corresponds to methylation cluster 2. Our database now contains methylation array data of 194 choroid plexus tumors (78 CPP, 44 aCPP and 72 CPC). In line with earlier results [33], CPP and aCPP branch into all three methylation classes, while CPC cases are uniformly associated with methylation class pediatric B (Fig. 1), emphasizing that current DNA methylation-based classification is not yet providing additional molecular layers for CPCs. Interestingly, a recent study has suggested that novel subclasses among patients with CPC may emerge [40], but these results will need to be validated in larger cohorts before they can be employed in a clinical setting.

## Treatment and trials

Treatment of CPTs may include surgery, chemotherapy and radiation. Given the rarity of CPTs, prospective clinical trials have been difficult, and treatment has often been based on results from small case series. Complete surgical resection has been shown to be curative for CPPs as well as aCPPs in most cases with no need for adjuvant therapy [14, 41]. Management of incompletely resected aCPPs is less clear, however infants with aCPP generally have a higher event-free survival than older children with aCPP independent of adjuvant therapy, hence therapy de-esclation may be warranted in low-risk subgroups.

While complete surgical resection is usually curative in CPPs, the best treatment modalities for CPC remain elusive and the prognosis remains poor. Complete surgical resection is desirable in CPC and has been shown to improve survival but is difficult to achieve upfront due to its infiltrative nature and high vascularity. Radiation therapy has been shown to be effective in the treatment of completely resected CPC patients [42] with 5-year PFS of 68% for patients having received irradiation compared to 16% for patients who did not. However, given the fact that the median age at diagnosis for CPC patients is 12 months with > 50% of tumors arising in the supratentorial location, a significant number associated with metastatic disease, and 50% of cases harboring a TP53 mutation, use of craniospinal irradiation would result not only in significant long-term neurocognitive and neuroendocrine sequelae but also an increased risk for secondary neoplasms.

Due to the small number of patients enrolled on infant protocols with CPC, there is limited data; however, chemotherapy does appear to have a beneficial effect in most series. Multiple clinical trials have used either conventional-dose or high-dose chemotherapy in in infants and young children with malignant brain tumors with an attempt to either avoid or delay radiation therapy. One of the initial trials using conventional-dose chemotherapy was conducted by the Pediatric Oncology Group (POG), deemed “Baby POG,” that opened in 1986 [43]. Five CPC patients were enrolled on that study; however, survival data were not presented. Children’s Cancer Group study, CCG9921, randomly assigned children < 3 years of age with malignant brain tumors between two chemotherapy regimens following surgery with an attempt to delay irradiation. Nine CPC patients were enrolled, of which seven had PD; five within the first year with a 3-year PFS of 33 + 16% and 3-year OS rate of 63 + 17%. UKCCSG/SIOP CNS 9204 trial utilized intensive conventional-dose chemotherapy schedule and included 15 patients with CPC. Eleven of 15 patients progressed on chemotherapy and died with a median time to progression of 0.46 years (range: 0.07–1.13 years). Only two patients had a complete surgical resection. The 5-year OS rate was 26.7% (CI 8.3–49.6) on this study [44]. In the Canadian Pediatric Brain Tumor Consortium experience [13] reported on 16 children < 3 years old with CPC, of which 14 had adjuvant conventional dose chemotherapy. Ten of 14 patients developed PD at a median of 10 months with a 5-year OS rate of 31 + 13.2%. The same group also reported on 17 children with CPC treated at a single institution, of which 12 children received adjuvant chemotherapy with ICE (ifosfamide, carboplatin, and etoposide) followed by second-look surgery. The number of cases with gross total resection increased from 2 to 9, resulting in a 5-year PFS and OS of 53 + 16% and 74.1 + 12.9%, respectively, for children treated with a curative intent without irradiation. The first prospective clinical trial for patients with CPTs was CPT-SIOP-2000 study that included maximal surgical resection followed by observation for patients with CPP or completely resected aCPP and adjuvant chemotherapy (etoposide, vincristine, and either carboplatin or cyclophosphamide) for patients with CPC, metastases or incompletely resected aCPP. Patients > 3 years old were treated with irradiation after the second cycle of chemotherapy [14]. For 92 of 106 eligible patients (42 with CPP, 30 with aCPP and 34 with CPC) treated according to the study protocol, 5-year EFS and OS probability rates were 92% and 100% for CPP, 83% and 89% for aCPP, and 28% and 36% for CPC patients, respectively.

Intensive induction chemotherapy followed by myelosuppressive high-dose chemotherapy (HDCTx) and autologous hematopoietic stem cell rescue (AuHCR) is routinely employed to treat infants and young children with malignant brain tumors in order to avoid cranial irradiation. Three sequential “Head Start” (I, II, and III) clinical trials, conducted between 1991 and 2009, enrolled 12 children with CPC and reported 5-year PFS and OS rates of 38% and 62%, respectively, with 5 children surviving irradiation-free at 29, 43, 61, 66, and 89 months from diagnosis. CCG99703, a Children’s Oncology Group study using a similar approach, enrolled four patients with CPC, of which two children were long-term survivors, one of whom had received irradiation at recurrence [45].

In a multi-institutional retrospective analysis, Tabori et al. stratified 36 CPCs by *TP53* mutation status and showed that 50% of CPC patients with *TP53* mutation had a 5-year OS rate of 0% as compared to 82 + 9% (without irradiation) for CPC patients with *TP53* wild-type [19], suggesting that biology of these tumors might have a larger influence on long-term survival rather than the intensity of treatment.

## Summary and future directions

DNA methylation profiling of choroid plexus tumors has revealed three epigenetically distinct and clinically relevant molecular subgroups. Epigenetic profiling will hopefully aid our understanding of choroid plexus tumor biology, can be used for the identification of patients at risk of recurrence and is expected to play an important role for treatment stratification and patient management in the context of future clinical trials. An integrative risk score of age and methylation cluster will be particularly helpful for aCPP. For the LFS cohort of CPC, a large co-operative group trial will have to assess the role of HDCT vs. conventional irradiation-free and alkylator-sparing therapy.
